# Virtue and medical ethics education

**DOI:** 10.1186/s13010-021-00100-2

**Published:** 2021-05-27

**Authors:** Will Lyon

**Affiliations:** Medical College of Wisconsin Affiliated Hospitals, 8701 W Watertown Plank Road, Milwaukee, WI 53226 USA

## Abstract

The traditional structure of medical school curriculum in the United States consists of 2 years of pre-clinical study followed by 2 years of clinical rotations. In this essay, I propose that this curricular approach stems from the understanding that medicine is both a science, or a body of knowledge, as well as an art, or a craft that is practiced. I then argue that this distinction between science and art is also relevant to the field of medical ethics, and that this should be reflected in ethics curriculum in medical education. I introduce and argue for virtue ethics as the best opportunity for introducing practical ethical knowledge to medical trainees.

There is a saying repeated so often in medical education that it has almost become a platitude. This is the saying that medicine is both an art and science. This does not mean that only a portion of the study and practice of medicine is within the grasp of empiric knowledge, and that the rest is up to intuition or guesswork. Rather, it refers to two distinct ways in which medicine must be approached. On the one hand, it is a science, or a body of knowledge to be learned. On the other hand, medicine is an art or craft that must be practiced and honed. This is an important distinction that applies to many pursuits: the distinction between knowledge and craft, or between theory and practice.

This distinction is reflected in the traditional medical school curriculum, divided into 2 years of intensive study of the scientific principles underlying medical practice, followed by 2 years of immersion in a variety of clinical environments and medical specialties. While this structure cleanly delineates medical theory and medical practice as separate areas of study, clinicians and medical trainees know from experience that the neat boxes of theoretical knowledge and practical wisdom are often entangled. For me (and for most, I would guess), the latter half of medical school was an exercise in learning how to reconcile the theoretical knowledge that I had learned over the first 2 years of medical school with the practical knowledge that the physicians around me had gained over years of actually treating patients.

The situation seems different when it comes to how medical ethics is taught to medical students. This difference lies in the fact that medical ethics is taught mainly at the level of theory, without much formal practical experience later on. One popular way to teach medical ethics to medical students is to present different ethical principles that should be weighed in our clinical ethics decision making. While variations exist, the most commonly presented factors are patient autonomy, beneficence, nonmaleficence, and justice or fairness. These principles all represent important considerations, and using this method of analysis can help identify the relevant moral considerations. However, the problem with this “principlist” way of teaching ethics is that there is no good way to weigh these principles against each other, since they are each based on and measured by different factors. Medical students, trained by years of multiple choice exams to memorize and move on, memorize the principles, successfully replicate them on an exam question, and move on.

Besides the incommensurability of the four principles, another reason that this way of teaching ethics falls short is that ethics ultimately involves action and behavior. Alastair MacIntyre reminds us in *After Virtue* that morality was traditionally perceived as centered on character, “where a man’s character is nothing other than his set dispositions to behave systematically in one way rather than another, and to lead a particular kind of life” [1]. As Aristotle puts it in in *Nicomachean Ethics*, “we become just by doing just actions, temperate by doing temperate actions, brave by doing brave actions” [2]. For MacIntyre and Aristotle, morality is a matter of character, and, therefore, of behavior. I do not think that current medical ethics educators would deny this. However, by emphasizing practical morality and character, medical ethics can become a skill that students continue to master during their clinical years, rather than a discipline that feels overly theoretical and remote.

It is true that ethics is very frequently taught in a case-based format, and is even taught during the clinical years of medical school. However, in my experience, this curriculum is sparse and, if a clerkship chooses to include it, tends to consist of some lectures sprinkled into the regular clinical schedule. Further, one might object that students and trainees do learn practical ethics from the physicians training them, by the very fact that they observe them navigating moral dilemmas on a regular basis. This is true, but it is just as easy to learn vices this way as it is to learn virtues. Imagine, for example, a student who spends months with a physician who is lazy, often lies or hides the truth when dealing with patient or colleagues in order to save time, and documents history or physical findings that they did not actually obtain. The student is learning moral knowledge and possibly even developing habits from this clerkship, but they are not the habits that we want our doctors or colleagues to possess.

I’m not proposing that there should be a mandatory “medical ethics” practical clerkship for third and fourth year medical students (although I would have signed up for one). Rather, medical ethics should be introduced to medical students as involving both theory and practice, with reinforcing examples and didactics throughout all of medical school and ideally residency training. Understanding ethics in this way, medical students would be able to consciously hone their moral decision making on the wards, instead of remembering medical ethics as a series of abstract principles they memorized for an exam during their first 2 years of school. As I see it, teaching medical ethics in this way would involve employing the concept of virtue.

The word virtue can have a lot of misleading connotations in our culture. I intend to refer to virtue in the Aristotelian tradition: as a habitual disposition to choose what is good. Aristotle defines virtue in a person as “the disposition which renders him a good man and also which will cause him to perform his function well” [2]. In other words, virtue is a disposition or state which both improves the goodness of the person and causes them to succeed in their role. For a doctor, virtue leads to healing well. Considering human virtue more broadly, virtue is what allows us to carry out the purpose of the moral life, which Aristotle took to be action in accord with reason.

Approaches to ethics that rely on the concept of virtue were called into question by modern philosophy, and are only recently getting renewed consideration. As Edmund Pellegrino writes in *The Virtuous Physician and the Ethics of Medicine*, “we have lost consensus on a definition of virtue, and without moral consensus there is no vantage point from which to judge right and wrong” [3]. Among increasing disagreement about the goal toward which virtue should aim, virtue became “confused with conformity to the conventions of social and institutional life” [3], which is a dangerous way to approach ethics. Yet, as Pellegrino points out, no matter how hard we try to banish the concept of virtue from ethical theory, the concept is difficult to escape in practice. He writes, “we know there are people we can trust to temper self-interest, to be honest, truthful, faithful, or just, even in the face of the omnipresence of evil” [3], and that at the same time there are people we know who cannot be trusted to act well in most situations.

Pellegrino uses this intuition to point out something that has been increasingly recognized in recent years – that despite the importance of various moral principles and individual rights, we cannot avoid the fact that moral decision making will always involve personal character. He states that this is especially obvious in medical ethics, “where the vulnerability and dependence of the sick person forces him to trust not just in his rights but in the kind of person the physician *is*” [3]. Most physicians can think of a handful of their colleagues that they would want as their doctor were they to fall ill, and they could probably name some colleagues they would not trust with their care. This is based not only on how competent and smart the person is as a doctor, but on that doctor’s virtues – are they honest, trustworthy, compassionate, selfless, etc.? Most importantly, can we expect them to do the right thing even when doing so is difficult?

Already in today’s medical schools, we promote a sort of medical virtue. We do this by educating medical trainees and physicians to ground their decision making based not on what the majority of practitioners are doing, but rather on evidence and on the context of the specific patient’s situation. Educating students and trainees about evidence-based medicine and initiatives like the American Board of Internal Medicine’s *Choosing Wisely* are examples of this promotion of medical virtue [4]. These initiatives depend on clinicians that are able to navigate situational subtlety, learn from experience, and recognize the goal of specific tests and interventions. Clinicians exercise medical virtue when they choose actions that will best achieve the end of medicine (i.e. health). It is inspiring to be on the wards, or in clinic, with physicians who are able to overcome the situational pressures set up by convention, institutional demands, and unsuitable guidelines in order to better care for the patient.

Aristotle (the son of a physician) himself recognized the many similarities between medical practice and ethics, and drew numerous comparisons between the two in his writings. In the Nicomachean Ethics, he compares someone who knows about virtue but does not perform virtuous actions to “a sick person who listens attentively to the doctor but acts on none of his instructions” [2]. Just as communicating a diagnosis does not on its own begin the healing process (physiologically speaking), identifying ethical principles does not on its own fuel moral development. It is the actual administration of therapy that causes healing, and it is the putting of moral principles, especially the virtues, into practice that promotes right action.

Just as we should honor the wisdom and confidence that skillful medical practice requires, we should also promote moral virtue, to the extent that we can while respecting individual differences in conscience and worldview. As we defined above, virtue involves a habitual disposition to choose the good. Our pluralistic society allows for reasonable disagreement about what constitutes the good life. However, there are many goods that we consider valuable as medical professionals and as human beings, such as happiness, health, human life, relief of suffering when possible, and friendship.

Even if this is all true, why would teaching ethics under the framework of virtue be any more successful than our current strategies? Some empirical data about the effect that situational factors have on persons’ moral decision may support the importance of habit over theoretical knowledge. For example, Darley and Batson’s “Good Samaritan” experiment in 1973 sought to identify relevant factors underlying helping behavior [5]. They recreated a situation akin to the parable of the Good Samaritan as found in the Gospel of Luke, in which a man beset by robbers is left on the side of the road beaten and injured. A priest and a Levite both pass the injured man and cross to the other side of the road, while a Samaritan passing by stops and tends to the man. Darley and Batson recreated this scenario in an experimental set-up with students of Princeton Theological Seminary as their subjects. Each subject was told that he was going to give a talk in another building on campus that would be recorded. On their way to their talk, they would pass a victim collapsed in a doorway of a campus building. Darley and Batson sought to identify under what conditions the students would stop and help the victim.

Among the variables Darley and Batson looked at were religious and moral orientations (based on a questionnaire), the topic of the talk they were assigned to give (half were assigned to speak on the parable of the Good Samaritan, the others received a different topic), and how much of a hurry the subjects were in. Some subjects were told that they were late for their talk and should hurry, some that they would make it just in time, and the others that they would arrive early to their talk. The only variable that Darley and Batson found to influence willingness to help was how much of a hurry the subjects were in: 63% of those who were in no hurry stopped, while only 10% of those who were running late stopped to help [6].

Some have argued that this experiment and others show that moral behavior can be contingent on seemingly unimportant factors. In the face of this assumption, moral theorists have revisited virtue ethics as a way to overcome contingency and promote habitual behaviors that can withstand various situations. As I mentioned above, this is not unlike the way in which various physician groups encourage colleagues to order the right tests and the right treatment independent of conventionality, institutional, and social pressures. For example, just because your colleague prescribes antibiotics for all upper respiratory infections doesn’t mean that you should. We should encourage the same approach when it comes to physicians’ moral decision making. As MacIntyre puts it,

The virtues therefore are to be understood as those dispositions which will not only sustain practices and enable us to achieve the goods internal to practices, but which will also sustain us in the relevant kind of quest for the good, by enabling us to overcome the harms, dangers, temptations and distractions which we encounter, and which will furnish us with increasing self-knowledge and increasing knowledge of the good [1].

It is important here to re-emphasize that developing these dispositions to the good takes practice, like the development of other dispositions that medical education attempts to cultivate in students and trainees. After being introduced to the theory early in medical school, trainees spend years developing their medical intuitions and approaches to various situations, so that they might choose well by the time they are practicing on their own. For example, just as we promote regularly taking a good history and a thorough physical exam, we might help trainees develop the disposition to be honest with their patients and to be charitable even to difficult patients.

One of the biggest grievances I heard from fellow medical students about ethics lectures was that they seemed impractical and inapplicable, and they often left the sessions feeling that there was no right answer. While dedicating class time to ethical theory is vital to developing moral intuitions and frameworks, ethics education will continue to seem impractical to medical students unless it is practiced as an art in the clinics and on the wards. However interesting it may be to analyze complex ethical problems for their teaching value, students ultimately need to form the habit of choosing well. As Aristotle describes,

“the mass of mankind, instead of doing virtuous acts, have recourse to discussing virtue, and fancy that they are pursuing philosophy and that this will make them good men. In so doing they act like invalids who listen carefully to what the doctor says, but entirely neglect to carry out his prescriptions. That sort of philosophy will no more lead to a healthy state of soul than will the mode of treatment produce health of body” [2]

In other words, no amount of *talking* about virtue can make you virtuous, because virtue can only be developed through the habit of doing what is right. This method of teaching ethics, by working closely with students to develop good habits, also fits with student preferences. In a 2017 survey of fourth-year medical students, “positive role models” was listed as the preferred method for learning ethics [7].

Teaching practical ethics to medical students is already done on the wards and in the clinic, for example when a physician explains her approach to a particular ethical dilemma. However, students and trainees lack a coherent framework within which to understand these sporadic lessons in moral decision making. By introducing the theory, or science, of virtue to medical students earlier on (e.g. in the first 2 years), they will be better equipped to notice and practice the art of moral decision making. This need not preclude teaching medical students important medical ethical principles such as autonomy and non-maleficence, but rather would emphasize that they ought to practice becoming the type of physician that prudently applies these principles.

## Data Availability

Data sharing not applicable to this article as no datasets were generated or analysed during the current study.
